# Pyogenic Sacroiliitis due to Group A *Streptococcus* following Uncomplicated Pregnancy and Vaginal Delivery

**DOI:** 10.1155/2013/981474

**Published:** 2013-12-11

**Authors:** Yoon Sik Park, Alexander Michael Owen, Alan Maurice Adno, Jyothi Marry

**Affiliations:** Liverpool Hospital, Sydney, NSW 2170, Australia

## Abstract

*Background*. Although the incidence of pregnancy-associated pyogenic sacroiliitis is low, it is associated with significant morbidity and mortality. Timely diagnosis of the condition is challenging due to its nonspecific clinical features. *Case*. A 31-year-old primigravida had an uncomplicated pregnancy and labour. Postpartum, she developed persistent fever and debilitating hip pain on ambulation. White cell count was normal (7.3 × 10^9^/L) and C-reactive protein was elevated (468.4 mg/L). *Streptococcus pyogenes* was identified on vaginal swabs and blood cultures, and a pelvic magnetic resonance imaging scan revealed bilateral sacroiliitis. *Conclusion*. Pyogenic sacroiliitis is a potentially lethal cause of postpartum pain. It should be considered as a differential diagnosis even in low-risk women who present with debilitating pelvic pain in or around pregnancy, particularly when initial therapy appears unsuccessful.

## 1. Introduction

The incidence of pregnancy-associated sacroiliitis is low, with less than 20 cases reported in the literature, occurring either during pregnancy, in the puerperium, or postabortion [1]. Its pathophysiology may involve relaxation of the pelvic ligaments during pregnancy, resulting in increases in pelvic movements and thus microtrauma to joint surfaces; this makes them susceptible to any transient bacteraemia occurring in the context of pregnancy-induced immunosuppression [1–3]. As such, it is more common in injecting drug users and individuals with concomitant infections such as endocarditis or endometritis [1].

## 2. Case Presentation

A 31-year-old primigravida presented to the delivery suite in spontaneous labour at term and progressed to a normal vaginal delivery, facilitated by a right mediolateral episiotomy. She was previously well, had an uncomplicated pregnancy, and had screened negative for Group B *Streptococcus* in a third-trimester low vaginal/anal swab.

**Figure 1:**
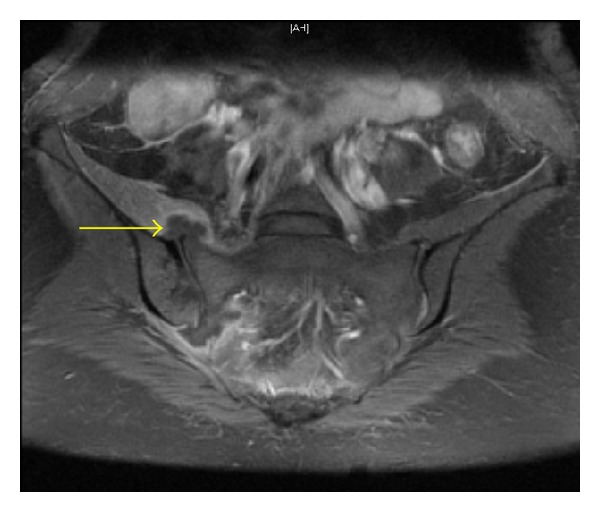
Bilateral sacroiliitis with right sacroiliac joint collection.

Two days postpartum, she reported mild perineal and right buttock pain without systemic symptoms. Physiotherapy and simple analgesics were prescribed. By the fourth day postpartum, however, she was unable to ambulate due to severe bilateral hip pain; she had a high-grade fever (39.9°C) and was haemodynamically unstable (heart rate 144/minute, respiratory rate 36/minute, and blood pressure 90/56 mmHg). Urine, blood, and perineal swabs were collected, revealing a normal white cell count (7.3 × 10^9^/L) and markedly elevated C-reactive protein (468.4 mg/L). A pelvic computed tomography (CT) scan showed no destructive bony lesions or intrauterine collection. Empiric intravenous antibiotic therapy (ceftriaxone and metronidazole) was instituted to cover for sepsis presumed to have a perineal source, and she was transferred to the intensive care unit for closer monitoring. An examination under anaesthesia revealed no evidence of a perineal abscess. By the day sixth postpartum, blood cultures and a perineal wound swab had grown Group A *Streptococcus *(*pyogenes*), so her antibiotic regimen was rationalised to intravenous benzylpenicillin. However, she continued to have fever and debilitating hip pain on ambulation. Further cultures of urine, blood, and perineal swabs were negative; pelvic deep venous thrombosis was excluded by a duplex ultrasound. On the 12th day postpartum, she underwent a pelvic magnetic resonance imaging (MRI) scan, which revealed bilateral sacroiliitis with a right-sided sacroiliac joint collection (Figure 1). This was subsequently drained under CT guidance, yielding 1 mL of purulent fluid and effecting a dramatic improvement in the patient's symptoms. She was discharged home on the 15th day on a four-week course of intravenous benzylpenicillin, followed by a six-week course of oral amoxicillin.

## 3. Discussion

The major causative organisms are Group A and B *Streptococci* and *Staphylococcus* species [1, 4]. Group A *Streptococcus* (GAS) is particularly important owing to its mortality rate of 20–25%: a consequence of the toxic-shock-like syndrome triggered by its exotoxin [5]. GAS can also cause rapid bone and joint destruction resulting in chronic disability for survivors [5].

Although timely diagnosis of pyogenic sacroiliitis is critical, it presents a diagnostic challenge. Its symptoms are nonspecific—low back and buttock pain are common in the puerperium—and it can occur in women without identified risk factors, for whom the index of suspicion for this aetiology would be low. Moreover, one-third of patients with pyogenic sacroiliitis have a normal white cell count and are afebrile [1]. The most useful investigations for establishing this diagnosis are inflammatory markers, which are almost universally elevated, and pelvic MRI [1, 2].

## 4. Conclusion 

This case demonstrates an unusual but potentially lethal cause of postpartum pain complicating normal vaginal delivery. Although rare, a differential diagnosis of pyogenic sacroiliitis should be entertained even in low-risk women presenting with debilitating pelvic pain in or around pregnancy, particularly when initial therapy appears unsuccessful.
